# Initiating a district-based public–private mix to overcome tuberculosis missing cases in Indonesia: readiness to engage

**DOI:** 10.1186/s12913-022-07506-4

**Published:** 2022-01-26

**Authors:** Deni Kurniadi Sunjaya, Cindra Paskaria, Dewi Marhaeni Diah Herawati, Meisera Pramayanti, Rini Riani, Ida Parwati

**Affiliations:** 1 Department of Public Health, Faculty of Medicine, Universitas Padjadjaran, Jalan Eyckman No 38, Bandung, West Java 40161 Indonesia; 2 Department of Public Health, Faculty of Medicine, Maranatha Christian University, Bandung, Indonesia; 3 Post Graduate Program, Faculty of Medicine, University of Padjadjaran, Bandung, Indonesia; 4 District Health Office, District of Purwakarta, Purwakarta, Indonesia; 5 District Health Office, City of Bandung, Bandung, Indonesia; 6 Departement of Clinical Pathology, Faculty of Medicine, Universitas Padjadjaran; Dr. Hasan Sadikin General Hospital, Bandung, 40161 Indonesia

**Keywords:** District-based public–private mix, Readiness to engage, Tuberculosis

## Abstract

**Background:**

District-based *public****–****private mix* (DPPM) is a variant of a relatively new PPM strategy of addressing missing cases in the tuberculosis (TB) care cascade in Indonesia. We aimed to determine the readiness of various stakeholders to engage in implementing the DPPM strategy.

**Methods:**

The research design was sequential exploratory mixed methods. A qualitative study in the first stage was carried out through in-depth interviews, FGD and study documents. Data were analyzed through coding, categorizing, pattern matching and theorizing. The second stage was a survey conducted using instruments built in the first stage. Data were analyzed using Rasch modeling and logistic regression.

**Results:**

District TB case detection rate (CDR) has improved from 35% (2018) to 104% (2019). The contribution of private hospitals has increased considerably. However, there were almost none from the private primary healthcare facilities. The substantive theory generated indicates that awareness and concern of the TB problem, TB program comprehension and involvement, and institutional support are behind the readiness of facilities to engage the TB program (the readiness to engage). The measurement results indicate the significant correlation of all dimensions on readiness to engage. Concern of the TB problem and institutional support are variables that influence readiness to engage (*p* < 0.05).

**Conclusions:**

Engaging private and public facility stakeholders is a challenge for local government. Intervention is through a personalized approach, encourages institutional support of health facilities for the TB program and system approach.

## Background

Indonesia, with the fourth largest population, in the world is ranked as the second of the eight countries that contribute to global tuberculosis. Those countries are India (26%), Indonesia (8.5%), China (8.4%), Philippines (6%), Pakistan (5.7%), Nigeria (4.4%), Bangladesh (3.6%) and South Africa (3.6%) [1, 2]. Tuberculosis (TB) is one of the top 10 causes of death in the world [2].

The COVID-19 pandemic situation, where the focus of attention is, has resulted in the neglected of the tuberculosis control program in Indonesia. This should be of the government’s serious concern and attention. Tuberculosis control is an essential service that must not be abandoned [3].

The World Health Organization (WHO) global policy for TB control is to involve all service providers through a Public**–**Private Mix (PPM) approach [4]. The goal of PPM is to improve case detection and treatment success [5, 6] which contributed to the missing cases. The target providers are not only private and corporate sector (hospitals or institutions, private practitioners), and voluntary sector (non government organization or community-based organizations), but also public sector itself (many types of public providers such as general and speciality hospitals, teaching hospitals, prisons, military-owned providers and others who have not joined the program [7]..

This policy has also been adopted by the Government of Indonesia. Research evidence in India and Myanmar indicates that PPM strengthens TB care and control [8, 9]. However, according to Wells et al., and Lei et al., the PPM TB approach is considered to be ineffective because it is very contextual, especially for financing and governance issues [5, 10].

The Indonesian health care system has long been formed from public and private providers [11]. In 2020, there are 27,443 providers for primary health care (PHC), of which 63% are privately owned. For secondary and tertiary health care, there are 2449 hospitals, of which 59% are also privately owned [12]. Nationally, most patients use public facilities. However, private providers are mostly located in urban areas and on the islands of Java and Sumatra [11].

Since 2014, private growth is getting stronger in line with the implementation of health financing reform into the form of social health insurance as National Health Insurance programme (NHI) [11, 13]. After sixth year of NHI implementation, 56% of private providers at the primary service level (clinics, independent practicing doctors) has been in collaboration with Social Security Agency for Health (SSA-H), the administer of NHI; while at the secondary service level, private providers (hospitals) are 56.3%, which are also higher than public providers [12]. NHI has reached 222.4 million people or 82% of the population in 2020. However, there are still out of pocket as much as 32% [14], not all patients who visit private providers have insurance or use their NHI. NHI members can choose and it is possible to switch between public and private providers.

The PPM TB approach in Indonesia has not yielded good results. According to WHO, Indonesia is one of the countries with case detection rate (CDR) under the 70% target [15]. One of the causes of the low CDR is the under-reporting of diagnosed TB cases, especially by the private sector [15]. These results are in line with the findings of Reviono et al., who stated that the CDR in Central Java Province of Indonesia before the implementation of PPM was very low. After the implementation of PPM, CDR has increased but did not reach the target, as it was still below 60% [16]. PPM implementation is based on not only the number of facilities involved but also other factors such as cessation of TB funding and staff’s tours of duty [16].

Missing TB cases in Indonesia remain high. This picture can be seen from the results of a TB inventory study in 2017, which found that there were 310,000 TB cases (44%) that had not been reported and 310,000 cases (30.4%) that had not been detected [17]. The research conducted by Febriyeni et al. had almost the same results as those of an inventory study in which the most missing TB cases occurred in private services compared to government [18]. Underdiagnosis mostly occur in government services, and under-reporting mostly occur in private services [18]. Private providers have contributed to TB management by 42%, but only 9% for case notifications [19]. The low achievement of CDR and high TB missing cases in Indonesia is likely due to low engagement of private health services.

In a decentralized government system such as Indonesia, the responsibility for the health sector is delegated to districts/cities. In 2016, the Indonesian government renewed the PPM strategy through the District-based Public–Private Mix (DPPM) [20]. DPPM is a tuberculosis service network in one district/city that involves all public and private health facilities, and coordinated by the District/City Health Office (DHO) [21]. The aim of the DPPM, similiar to PPM, is to drive all healthcare facilities that handle TB patient to participate in the network, so that all TB patients can be found and treated according to standards and recorded in the national TB program information system [21]. One form of DPPM activity is the network of public and private health service facilities.

Public providers have the advantage of getting the TB drug supply facilitated by the government, in which the cost of purchasing drugs from private providers is borne by the patient. The cost burden on these patients often leads to dropouts, without the patient being traceable. On the other hand, public PHC (namely Puskesmas), which are under responsible of DHO, has human resources to outreach cases to the community, when there is a loss to follow-up. DHO becomes the conductor for all public and private assisted by a centralized recording and reporting system. The network enables the private sector, which has great potential to manage TB patients, at the same time taking advantage of facilitation from the public, including notification of TB cases, thereby reducing missing cases. Thus, networking between the public and private can reduce missing cases and improve the performance of the national TB program.

In 2020, the implementation of the DPPM was far from satisfaction. The new implementation guidelines were published in 2019, even though the DPPM policy was set in 2016. As a result, the cities/districts have not been able to implement the policy. In addition, the COVID-19 pandemic has disrupted all efforts, so that all programs and activities have been so far hampered and have not been able to run. In collaboration with the DHO, the researchers intend to carry out the operationalization of the DPPM as a strategy in tuberculosis control improvement.

Private sector engagement is very important to achieve the health goals in various programs [22]. However, the willingness and the readiness of the private sector to engage in health program, including in the tuberculosis control program, is not known yet. How readiness to engage in the TB DPPM strategy is important to explore. This readiness includes private institutional providers as well as health personnel in it. Therefore, before initiating the implementation of TB DPPM, it is necessary to explore this engagement readiness. Stakeholders’ engagement is the key to the realization of a service network within DPPM. Key stakeholders are health personnel, managers and provider owners involved in TB services, both in the private and public health services. We aimed to determine the readiness of various stakeholders to engage in implementing the DPPM strategy. Readiness means willingness, motivation, eager to act DPPM engagement. We describe in a more detailed objective as follows: 1). to explore excisting public and private engagement in TB program at a district sample; 2). to construct aspects of readiness to engage; dan 3). To analyze factors influencing readiness to engage in DPPM TB.

## Method

The district of Purwakarta, the research site, has initiated the implementation of DPPM by gathering stakeholders in a series of meetings. Researchers support DHO in managing change by providing assistance, facilitating meetings and formulating DPPM according to local conditions. Activities were carried out just before the COVID-19 pandemic occurred. This research is the initial stage of action research on the development of the DPPM implementation model [23, 24].

### Study context

This research was conducted in a district on the island of Java with a population of nearly 1 million. Public health facilities consist of 1 District General Hospital and 20 public Primary Health Care (*Puskesmas*), which have work areas’ responsibility in each subdistrict. Private health facilities are much more numerous, consisting of private hospitals (11), private clinics (92) and independent practicing doctors or private physicians (57) and private pharmacies (67).

### Study design

The research design was mixed methods with sequential exploratory mixed methods [24] and pragmatism paradigm [25]. Stage 1 is a qualitative study using indepth interview and focus group discussion methods, as a triangulation tools to increase trustworthiness, and the grounded theory approach [26]. The resulting substantive theory is used to develop quantitative research instruments. In addition, researchers conducted a document study of available data in the DHO information system. In stage 2, a quantitative study, measurements were carried out using the survey method. Then, statistical inference can be performed on the variables found in stage 1.

This research is an initial stage of a multiyear research. Data collection activities up to data analysis were carried out from January to July 2020. This design is used to understand the problem deeper, so that a DPPM implementation strategy can be developed according to the circumstances.

The study population was healthcare professionals working in public and private healthcare facilities. The inclusion criteria were: doctors, nurses, midwives, health service managers and program holders as seen in Table 1. Their functions include a person in charge of medical, a program manager and an institutional owner. Research subjects in qualitative research were selected purposively and successfully recruited 60 respondents for in depth interview and 44 respondents for 2 Focus Group Discussions (FGD). The informants in the two FGDs were the same: health workers from the public and private sectors as shown in Table 1. Such number of participants is selected so that the data can represent the situation in 20 sub-districts and the perspectives of public and private sectors. In addition, it is expected to increase the trustworthiness of the research results. Likewise, the need for item development for research instruments can be better facilitated. Quantitative data was collected from 31 respondents by non probability sampling.

**Table 1:** Characteristic of the respondents

	In depth interview		Focus Group Discussion	
	N	Percentage (%)	N	Percentage (%)
Gender				
Male	19	31.7	15	34.1
Female	41	68.3	29	65.9
Profession				
Doctor	34	56.7	30	68.2
Nurse/ midwive	10	16.7	6	13.6
TB Programmer	5	8.3	5	11.4
Others	11	18.3	3	6.8
Sector				
Public	30	50	21	47.7
Private	30	50	23	52.3
Institution				
Public Primary Health Care (PHC)	20	33.3	19	43.2
Hospital	7	11.7	7	15.9
Private phisicians (PPs)	10	16.7	10	22.7
Clinic (private PHC)	8	13.3	6	13.6
Pharmacy	2	3.3	2	4.5
District Health Office	8	13.3		
Profesional Organisation	5	8.3		

### Data collection

The study document was obtained from DHO’s tuberculosis report. In depth interviews were conducted on research subjects by 20 trained researchers and research assistants, while the FGDs was facilitated by the researcher themself. Based on in-depth interviews, and FGDs a research instrument was built. Then, by using this instrument, variables in the quantitative subject group were measured. Quantitative data were collected from respondents who were gathered in the initiation of DPPM TB implementation meeting. The respondents consist of targeted public and private provider to involve in DPPM TB.

### Data analysis

Secondary data (from documents) were analyzed using descriptive statistics. All data from indepth interview and FGD were transcribed directly. Transcription was processed through a reduction, and the coding and categorizing processes were then carried out. Six categories were generated and become the concepts built in this research. Pattern matching to these concepts and theorizing was carried out to produce a substantive theory [27]. Trustworthiness was obtained through triangulation of sources (informants) and methods and peer debriefing. The results of qualitative data analysis are used for the next stage, developing quantitative research instruments. In addition, the theme of the resulting concept is also drawn up, and is described along with its quotations.

Based on the substantive theory, a research instrument (questionnaire) was built consisting of 6 dimensions and 31 items. These dimensions are derived from the concepts (theory) that have been built, while items are developed from the resulting coding. Each dimension of this instrument consisted of 3 to 8 items. This number of items per variable is based on the resulting coding and testing using Rasch modeling. The type of questionnaire items were statements and answer which applying the Likert scale.

The research instrument or questionnaire was then used to collect quantitative data through a survey from 31 stakeholders. Quantitative data, which were ordinal, then were transformed using Rasch modeling [28] into numerical data on the six variables. The reliability of the instrument was 0.91; separation, 3.25; Cronbach’s alpha, 0.86. Raw variance explained by measure was 41.4%; unexplained variance in the first contrast was 15.7% (eigen value: 5.33). Then, the correlation test and linear regression analysis were carried out between the independent variables, namely: awareness, comprehension, concern, involvement and institutional support, and the dependent variable, namely readiness to engage.

### Ethical consideration

In both qualitative and quantitative research, written informed consent is obtained from the subject.

## Result

### Excisting public and private engagement: the problem

Case detection rate (CDR) of the district has increase from 35% (year of 2018) to 104.42% in the year of 2019. Private hospitals contribute 3 times as much as public hospitals. So, both hospitals could achieve more than a half of district target. The contribution of private hospitals is relatively high and has changed positively from the previous year. This is a major advance in the public-private mix.

**Fig. 1:**
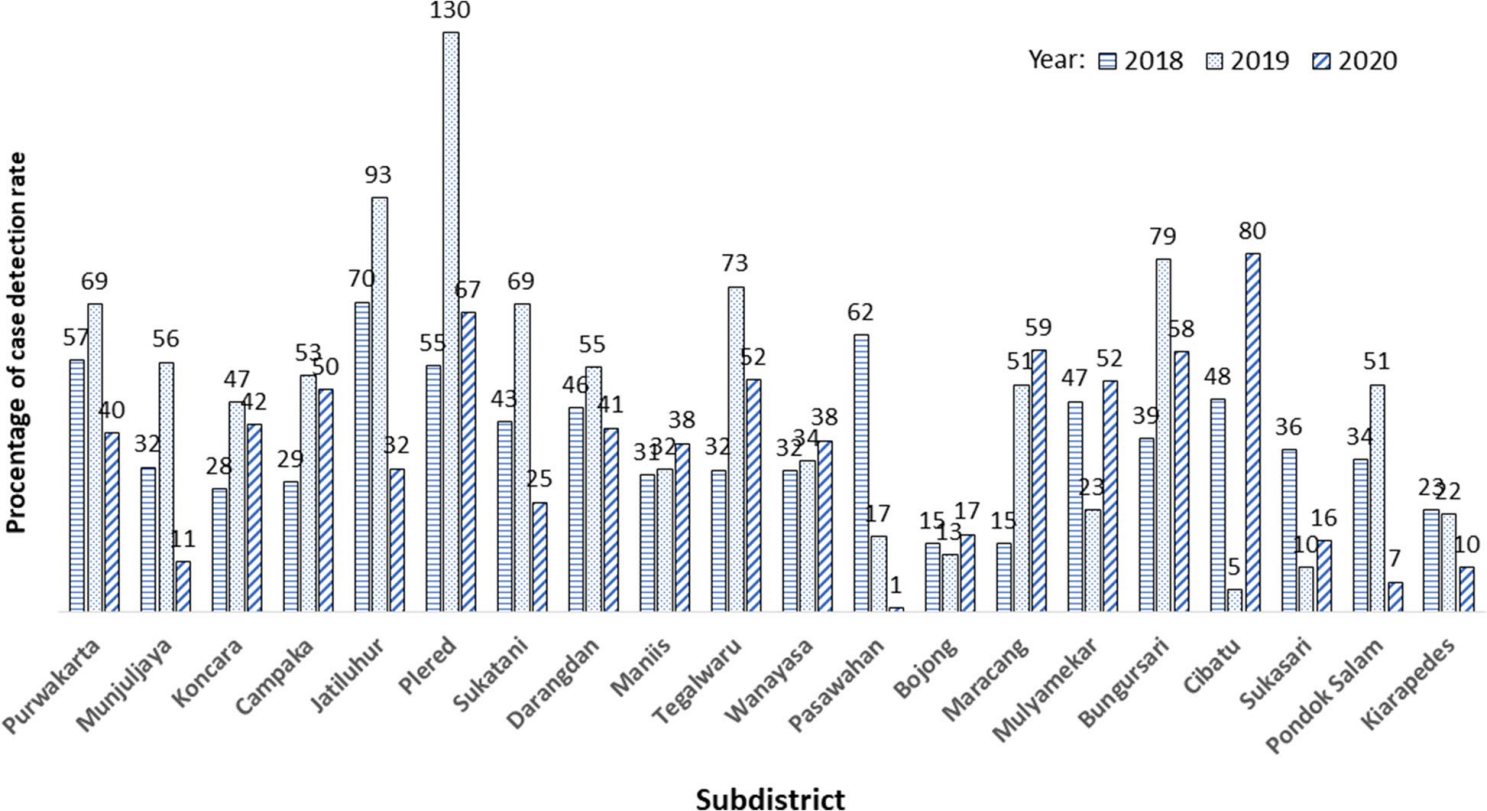
CDR in 2018–2020 by subdistrict/public PHC (%)

Achievements in 2020, during the pandemic, appear to be decreasing, but records are not complete until the end of the year, meanwhile it cannot be concluded yet. The achievement of CDR in the sub-district (under *Puskesmas* responsibility) was almost half of district target on average as can be seen in Fig. 1. However, there is almost no contribution from PHC facilities.

Hospital outreach to TB cases is very limited as a result of its function as a referral facility. On the other hand, PHCs (public or private) are actually closer to the community. The performance of PHC in TB case outreach can be further encouraged in increasing access for TB sufferers to health facilities.

As stated above, public PHCs (Puskesmas) contributed almost half of the district’s CDR achievement. Notably, in this pandemic situation, 30% of the sub-districts have increased the CDR, whereas others’ CDR of this Public PHCs has drastically dropped. The assumptions that arise from program holders are population factors and turnover of subdistrict case managers. This phenomenon supports the suspicion that many TB suspects do not have access to healthcare services. By contrast, the COVID-19 pandemic situation has affected the TB control program. Case detection and reporting activities from private hospitals are increasing every year, but almost none from private primary healthcare facilities.

In this district, the role of the private sector is illustrated by the CDR donated by private hospitals. However, the role of private PHC is almost non-existent (Table 2). This situation occurs because the private PHC does not manage TB patients. They tend to refer TB patients to hospitals and health centers. In addition, there are cases where private PHC does not report the cases found. This means that many missing cases still occur.

**Table 2:** Engagement of public and private hospital and PHC

	Public	Private
Hospital	Already engage; passive case finding	Already engage (2019); passive case finding; Increase in reporting
PHC	Already engage; active case finding; Variation of performance between PHC facilities	Not yet engage; low case reporting; Tend to refer cases.

Hospitals tend to be passive in accepting and managing TB patients, because of their given role as referral facilities. Considering that the problem of TB is access to health services, the role of PHC can actually increase access to services better than hospitals, because of their place as gate keeper, the main entrance, in the health system.

Indeed, the Puskesmas have detected cases with variations, there are Puskesmas whose performance exceeds the target, some are still far from the target. The phenomenon of Puskesmas having exceeded the target performance proves that cases in the community are far higher than the target set by the government. This means that more intensive case detection and tracing efforts are needed. This includes the role of a private PHC, the private clinics and phycisians as well.

Reflect on the results of the analysis above, engaging private PHC facilities in increasing CDR is absolutely essential. Several reasons, which were derived from qualitative analysis, why doctors in private facilities are reluctant or hesitant to treat or manage tuberculosis patients are: 1) increase the risk of transmission at the practice site; 2) high drug costs; 3) treatment complexity; 4) human resource limitations; and 5) feeling excluded. We include some quotes below.

*“…the risk of transmission lies with us as a private practice. Because the risk is the patient will mingle with other patients. Meanwhile, if we practice in private, we have limited resources*, *such as space, human ressources, and so on…” (clinic doctor, male)*

*“………if I find a TB suspect, he will immediately be referred, because there is no support. If you want to give the therapy, the medicine is not available. It is too expensive to prescribe outside. For 1 package, it's quite expensive. Especially for patients who can not afford to buy outside…” (clinic doctor, female)*

*“…TB does develop, the treatment takes a long time. There's a lot of medicine, after all, the medicines are separated. So, there are also cases that drop out. So, I don't want this to happen. Therefore, it is better to refer cases. (PPs, female)..”*

Then, we explored stakeholders’ perception regarding the possibility of engagement from private PHCs into the DPPM strategy.

### Aspects of readiness to engage

**Fig. 2:**
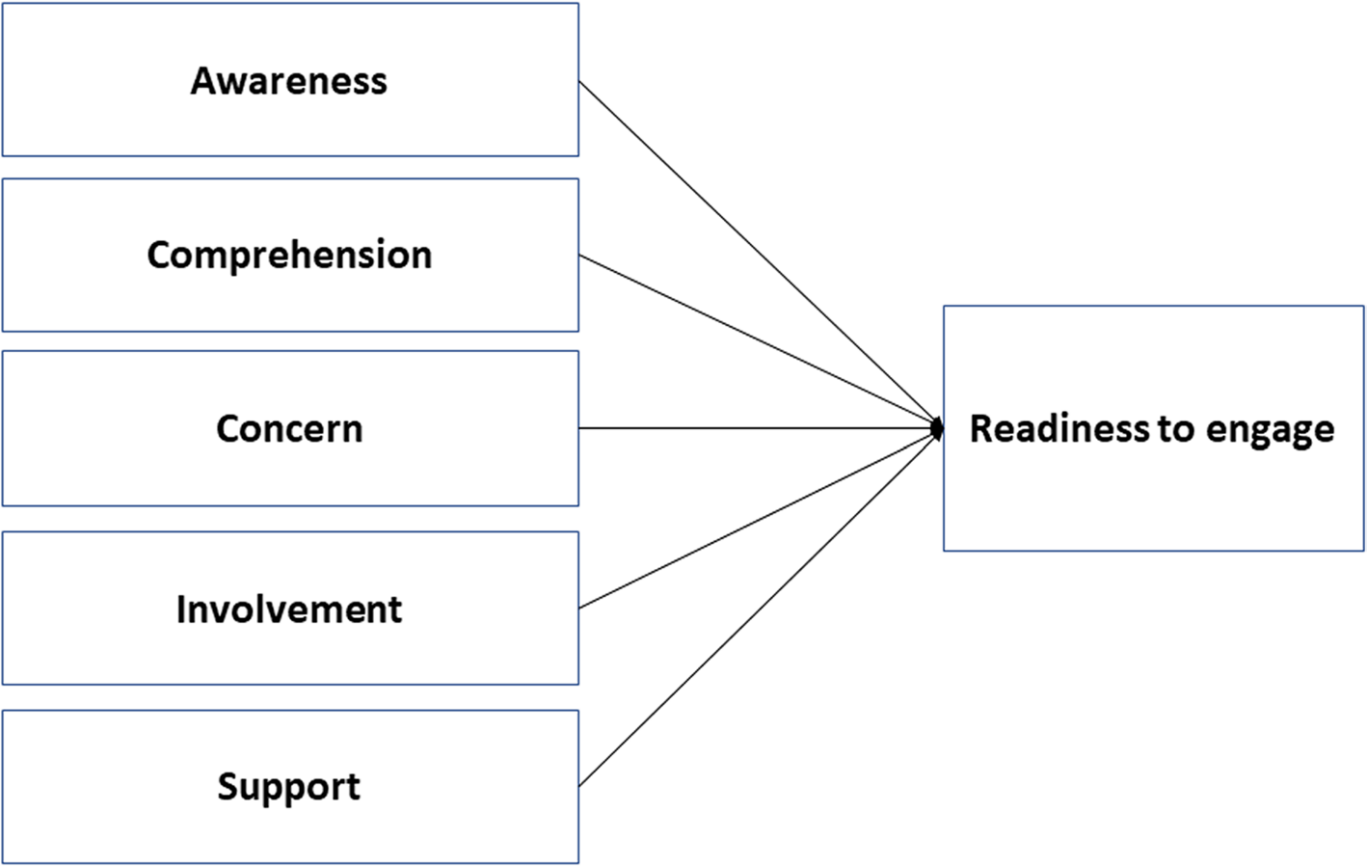
Factors influence the readiness to engage

Based on the results of the qualitative study analysis, a substantive theory has been successfully developed as illustrated in the Fig. 2. DPPM as strengthening of the PPM strategy really requires the engagement of private and public health facilities to be involved in it. Before this engagement occurs formally, it is necessary to know factors of the involved individuals’ readiness to engage.

Readiness to engage, as shown in Fig. 2, depends on individual and ecosystem (external) factors. Individual factors consist of an attitude of awareness and concern for the tuberculosis problem; comprehension of drug management, the tuberculosis control program and PPM management; and involvement in the tuberculosis control program. Institutional support is an ecosystem (external) factor for individual doctors and health workers to engage in tuberculosis control programs and DPPM.

So far, private physicians (PPs) have mostly referred TB patients or suspected TB patients, because it is risky for their place of practice/clinic. However, they are willing to treat if the medicine is provided by the government freely. PPs are willing to record and report if requested. They are also willing to be trained when requested. However, they objected to tracking cases. One respondent suggested coercive action through regulation:13, paragraph 3

*"……maybe it needs to be made a regulation that forces the private sector..."* (PP, male).

### Awareness of the problem

Respondents observed that there are still many TB suspects who do not have access to health services. The respondent awareness of tuberculosis cases, thinking about these problems and having views that should be carried out by the related parties shows awareness of th problem.

*“…My worker has relatives, and all of them are positive for TB. So it means that there are many possibilities in that area, it's just that maybe the work area of the puskesmas has not yet reached there...* "(PP, male)

Being aware of the tuberculosis problem in their area will encourage health workers to try to handle them according to their competence and authority.

### Program involvement

PPs have not collaborated with the government (DHO) and feel they have never been invited to collaborate by the Puskesmas. They have not yet been exposed to information systems for tuberculosis management:

*"... education to the community is mostly from the puskesmas program holders; in the private sector, I don't think I have heard either...."* (PP, female).

PPs are almost never involved in the program, as stated by a respondent.

*“………. I, as a personal doctor, am rarely around (exposed to the program). Most of the meetings with the Health Office are not convened by the doctors; most are by the TB administrators…. "* (PP, female)

Private hospitals have started to be involved in the TB program. However, it needs to be realized by the government that private doctors and clinics should be taken into account, given that patients usually come to primary care first.

*"…………The TB DOTS program is targeting now that it has entered private hospitals; maybe it would be nice now because there are more clinics than private hospitals, so this clinic is involved ……"* (PP, male)

### Program understanding/comprehension

The understanding of tuberculosis treatment management is not evenly distributed in every doctor, both public and private. Private doctors who have the opportunity to access the program can better understand and manage patients in line with government guidelines. The understanding of PPM is very low, not only in private doctors but also in public healthcare providers. Without good understanding, it is difficult for motivated individuals to become involved in government programs and strategies.

### Concern

Concern about TB problems in his area was illustrated by one of the private respondents:

*"... during the 7 years I was here, I saw that this TB doesn't go down...."* (PP, male)

Concerns about TB problems that have not been resolved are not only in the form of attitudes. This concern is the potential for action when the right situation and moment arrives. In this case, individuals who are concerned will get involved if invited by the public.

### Institutional support

Carrying out PPM requires enhancing patient management and program management skills (including recording and reporting). This requires additional human resource support and financing. If the institution that owns the health service is not supportive, then individuals who wish to be involved in the program cannot access public strategies and programs.

### Factors influencing readiness to engage

**Fig. 3:**
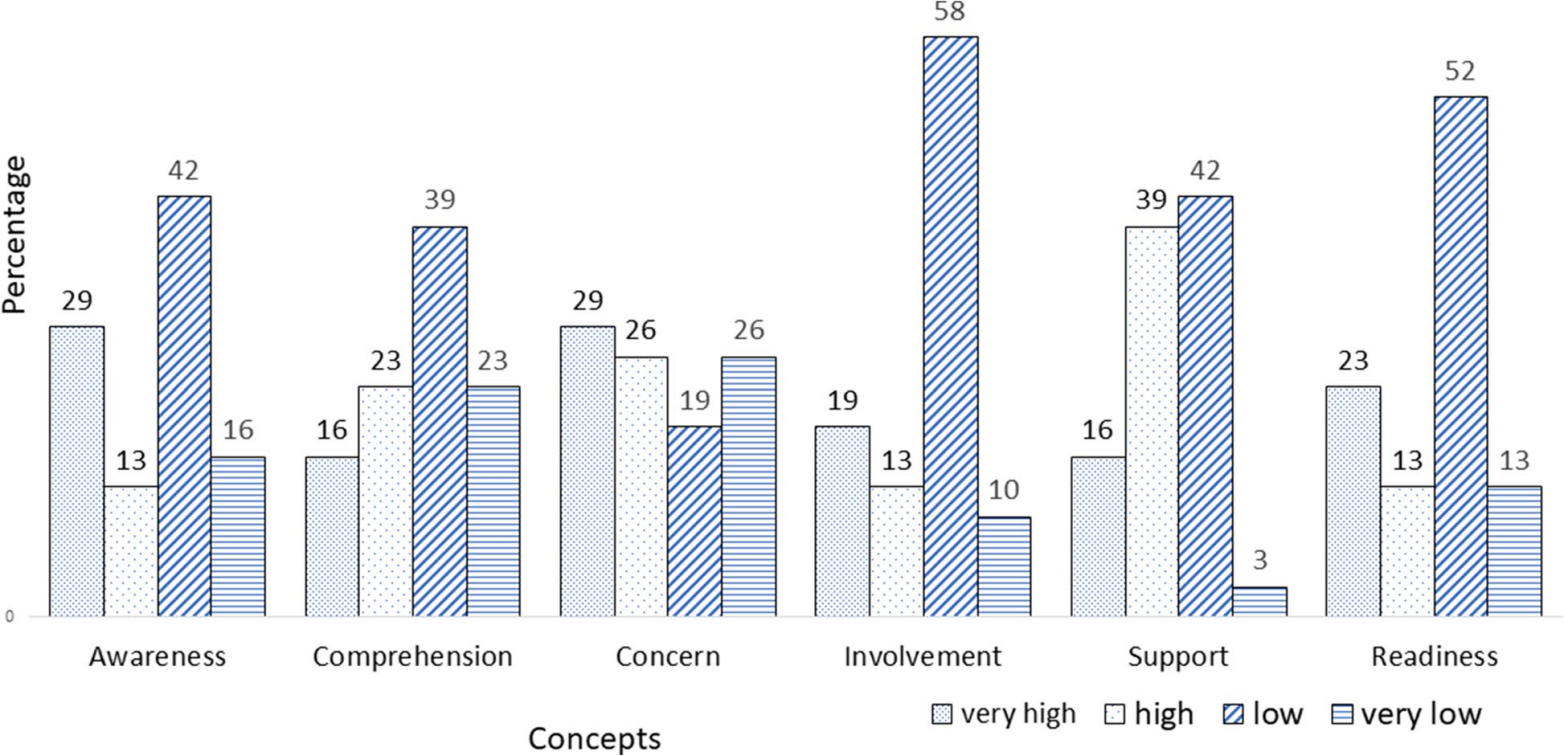
Readines to engage and its factors

Figure 3 illustrates the measurement results in a representative sample of private and public health facilities where the percentage of four levels out of six dimensions is seen in relation to the readiness of health facilities to be part of the DPPM strategy. Four dimensions, namely: awareness, program comprehension, concern, and program involvement, are those aspects that come from internal respondents. Meanwhile, institutional support is a factor that comes from outside the respondent.

The dimension of program involvement has the highest level below the mean (very low and low) (68%) followed by readiness to engage (65%). Circumstances factors describing a negative or less supportive trend. By contrast, it is interesting to follow the phenomenon where the most positive trends (high and high levels) are in the concern (55%) and awareness (42%) dimensions.

All dimensions have a tendency toward the right or a negative trend; this means that a portrait is less supportive of the occurrence of DPPM. The dimension of readiness to engage as the dependent variable also indicates a tendency of lack of support for the implementation of DPPM. This description is a challenge in building a DPPM implementation strategy.

Bivariate analysis indicated a correlation between readiness to action and awareness (r: 0.49; *p* = 0.02); comprehension (r: 0.518; *p* = 0.01), concern (r: 0.620; *p* = 0.00); involvement (r: 0.405; *p* = 0.012); and institutional support (r: 0.373; *p* = 0.019). However, in the regression analysis, there are only two dimensions that are significantly related to readiness to engage, namely concern and institutional support. So that we get Y = 0.205 + 0.239 X1 + 0.758 X2, where Y = readiness to engage, X1 = concern and X2 = institutional support (r^2^ adj. = 50.7%; *p* < 0.05).

The DHO as a dirigent of DPPM should encourage personnel in health facilities to be more concerned with TB problems. Equally important, local government encourages health facilities’ owners to support and facilitate professionals in their institution for tuberculosis services.

## Discussion

The potential of private sector in the DPPM strategy to address tuberculosis missing cases at the research site is quite large, but their involvement is still low. Initiating DPPM requires understanding their perspective on TB program engagement. The results show that the readiness of private providers to engage in tuberculosis control program depends on the following aspects: awareness, comprehension, concern, involvement and support. However, the dimensions of concern and institutional support that significantly affect the occurrence of readiness to engage at the research site.

One of the goals of the DPPM TB is to improve case detection. The document study indicates that the CDR in District Purwakarta, West Java Province, Indonesia in 2019 (before the COVID-19 pandemic) has increased, even exceeding 100% of the target. This can be explained by increasing of the involvement of several private hospitals in the TB program compared to the previous period. The phenomene is different from those of research conducted by Reviano et al., which was conducted in Central Java, where the CDR has not reached 60% [16]. Some of the patients may be from neighboring districts and are recorded in the system. This is possible, because patients are not prohibited from seeking treatment beyond the district administrative boundaries.

However, the excess percentage as mentioned above is relatively high. Another possibility that occurs is that the actual prevalence of TB in the community exceeds the targeted rate of 316 / 100,000 population. If this happens, it is necessary to strengthen the case finding of those who are unable to access, diagnosed and unregistered. They are the missing cases. On the other hand, the public PHC is hardly involved for reasons that should concern the DHO.

The substantive theory found from the stage 1 of this study indicates that the readiness to engage with DPPM occurs because there is awareness of TB problems, TB program involvement, TB program comprehension, concern of TB problems and institutional support for TB programs. Doctors in public and private services should have awareness of the TB problem. In fact, many doctors are not aware of the TB problem. A study in India found that patients who come to private facilities for TB care do not receive adequate care [29, 30]; there may be amplification of drug resistance [31]; and patients spend much money on inaccurate tests [32]. The conditions in India indicate that the awareness of private facilities on TB problems is still low.

In both the PPM and DPPM approaches, all health facilities are ideally involved in the TB program. However, the fact is that there are private doctors who have not been invited by the DHO to be involved in PPM or DPPM. The results of this study are in line with those of research in India, which states that the public sector is the main player and determines the terms and conditions, whereas the private sector plays a small role and must agree to these terms and conditions [33]; this condition causes private facilities to feel uncomfortable. However, the involvement of private facilities for TB programs is not solely due to incentives but rather the benefits that patients will receive from the TB program, and incentives are secondary [34].

This study found that the TB comprehension program about tuberculosis treatment management was not evenly distributed among doctors, both public and private. Private doctors with access to TB programs can better understand and manage patients in line with government guidelines. This is consistent with studies in India, where private practitioners have low knowledge of protocols for diagnosis and treatment of TB [35, 36].

The results of this study indicate that the concern in TB programs in the private and public sectors is still low regarding the high prevalence of TB in Indonesia. The results of this study are supported by a multicentric study in the cities of Delhi, Mumbai, and Patna, which found that 38% of pharmacies administered antibiotics (fluoroquinolones) or steroids to patients with TB symptoms, but there were no test results [37]. These drugs have the effect of delaying the diagnosis of TB; besides, fluoroquinolones are a drug regimen for the treatment of drug-resistant TB, so their use is of concern.

The results of this study on institutional support for TB programs found that implementing PPM requires improving patient management skills and program management (including recording and reporting). This requires additional human resource support and financing. This is in line with the opinion of Lei et al., who stated that cost is an important strategy in PPM, because the implementation of PPM requires a large amount of money [5].

The engagement program in DPPM TB requires the involvement of all stakeholders. According to Diamond, the involvement of all stakeholders is very important for sustainable development and resource management, improvement of the design and implementation of these activities and building of local understanding and ownership [38].

This study found that readiness to engage depends on individual and ecosystem factors. The results of this study are the same as Weiner’s opinion, that readiness to changes refers to the commitment to change organizational members and change the effectiveness of implementing change [39, 40] and is a multifaceted construct [41]. Weiner said that readiness to change depends on change valence, contextual factors and informational assessment [41].

According to Weiner, individuals in organizations that have low change preparation will refuse to make changes, whereas individuals in organizations that have high preparation for change will be happy to make changes, so that the implementation of change is successful [41]. Organizations can intervene with a regulatory approach, but this is not the case with individuals in the organization, so that individual readiness to change and be involved is very important.

According to Holt et al., organizational readiness is considered to be a major determinant of successful implementation and a mediator of the effectiveness of implementation interventions [42]. This is different from the findings of this study, where individual readiness is more important in achieving readiness to engage. According to Matthyesen and Haris, there is a relationship between readiness to changes and work engagement; a high level of work involvement will result in a high level of readiness to change [43].

Missing cases occurred in cases that did not have access to health services, were not registered, were not diagnosed and dropped out. PPM or DPPM will not mean much to solve the problem of tuberculosis missing cases if the strategy implementation is not managed properly. The long process from policy formulation (2016) to policy implementation (2019) indicates disruptive inaction due to poor management. The government’s intention to overcome this problem should be supported by change management, whose effectiveness can be improved by the role of academics.

WHO addressess 5 domains of engagement: public provision of services; policy and dialogue; information exchange; regulation and financing [44]. This study offers policy recommendation to increase and maintain readiness to engage which can be addressed to individuals, institutions or systems within the DPPM itself. The researchers think that the following recommendations are in line with WHO’s framework but appropriate to the context at the research site.

Interventions related to individuals (personalized approach) are: (1) increasing the number of popular scientific meetings related to tuberculosis for various healthcare professions, (2) routinely involving key stakeholders in formal tuberculosis program development meetings involving private and public health workers, and (3) extending disease management capacity building programs, such as training, to private human resources.

Interventions related to private institutions are: (1) annual regular meeting to invite leaders of private health service institutions and (2) regular visits DHO makes to the institution. Interventions related to DPPM as a whole (system) are: (1) building cloud-based information and communication media that easily connect public and private individuals and institutions for updating information and TB management, (2) developing local regulations that facilitate and support private sector involvement, and (3) increasing formal cooperation in a planned and continuous manner with individuals or institutions.

### Strength of the study

This study begins with qualitative exploration, so that it can produce readiness to engage variables with measurement items for each variable originating from the field itself. Inference made through analysis of survey results, is based on these exploratory stages.

### Limitation of the study

The number of quantitative samples is limited and is not randomized. Surveys are not intended to make representative generalizations. However, it is more of a management tool in compiling a situation analysis and developing strategies. Both the instrument and the measurement results are quite valid and reliable. However, the results of exploration and measurement are sufficient to lead district health management to take more evidence-based decisions and actions.

## Conclusions

The involvement of private PHC facilities in controlling tuberculosis, especially missing cases, has not yet occurred. Based on our analysis, the factors of Awareness and Concern of TB problem, TB Comprehension and Involvement and Institutional support program correlated with readiness to engage. However, two of them, the concern about the TB problem and institutional support has a significant effect. Interventions for DPPM implementation use individual, personalized, institutional and system approaches. Communication and information media between public and private health care facilities should be facilitated. Local regulations are built for the certainty and sustainability of DPPM.

## Data Availability

The datasets used and/or analyzed during the current study are available from the corresponding author on reasonable request.

## Abbreviations

CDR: Case Detection Rate
DHO: District Health Office
DPPM: District-based Public–Private Mix
DOTS: Directly Observed Treatment Shortcourse
TB: Tuberculosis
PCC: Patient Care Cascade
PHC: Primary Health Care
PP: Private Physician
PPM: Public–Private Mix
